# Brucellar Endocarditis of the Tricuspid Valve: A Case Report and Review of the Literature

**DOI:** 10.3390/pathogens13030239

**Published:** 2024-03-08

**Authors:** Evangelo Boumis, Pierangelo Chinello, Vincenzo Galati, Simone Topino, Francesca Gavaruzzi, Stefania Cicalini

**Affiliations:** Clinical Infectious Diseases Department, National Institute for Infectious Diseases “Lazzaro Spallanzani”, IRCCS, 00149 Rome, Italy; evangelo.boumis@inmi.it (E.B.); vincenzo.galati@inmi.it (V.G.); simone.topino@inmi.it (S.T.); francesca.gavaruzzi@inmi.it (F.G.); stefania.cicalini@inmi.it (S.C.)

**Keywords:** brucella, endocarditis, tricuspid valve, bacterial infections, foodborne infections, antibiotic treatment

## Abstract

Brucellar endocarditis is a rare entity commonly described as a severe disease associated with high mortality and generally requiring valve surgery for cure. Right-sided endocarditis, a very uncommon presentation of brucellosis, may be associated with a better prognosis. We describe the case of a 72-year-old woman admitted to our institution with a persistent fever and multiple pulmonary infiltrates. Transthoracic echocardiography and serologic tests led to the diagnosis of brucellar tricuspid endocarditis. The patient responded favorably to antibiotic treatment alone and did not need surgery. Prolonged antibiotic therapy with a combination of drugs active on intracellular microorganisms in the absence of surgical treatment could be effective in brucellar tricuspid endocarditis when the valve is not severely damaged.

## 1. Introduction

Brucellar endocarditis is a rare entity commonly described as a severe disease associated with high mortality and generally requiring valve surgery for cure. Right-sided endocarditis, a very uncommon presentation of brucellosis, may be associated with a better prognosis. We describe a case of brucellar tricuspid endocarditis that responded favorably to medical treatment alone and present a review of the literature.

## 2. Case Report

On 5 July, a 72-year-old woman was transferred to our institute with a diagnosis of persistent fever associated with multiple pulmonary infiltrates. Five months before, she had developed weakness and nocturnal sweats, followed four weeks later by fever with peaks over 39 °C. Her general practitioner prescribed various courses of unspecified oral antibiotics without benefit. She was admitted twice to her local hospital, where diagnostic imaging revealed a pulmonary infiltrate of the right lung base. She received different courses of antimicrobial therapy, including ceftriaxone, clarithromycin, and ceftazidime, and was eventually discharged with a diagnosis of slowly resolving right basal pneumonia. On 11 June, she was admitted to a regional hospital because of a continuing fever and dyspnea; a chest CT evidenced disseminated infiltrates in both lungs. Blood cultures were sterile; tests for tuberculosis on sputum, i.e., staining and culture, were negative; bronchoalveolar lavages for bacteria, fungi, and mycobacteria were negative. Transthoracic echocardiography revealed a thickening of the right coronary cuspid of the aortic valve, along with slight mitral and tricuspid insufficiency and signs of pulmonary hypertension (systolic pulmonary arterial pressure of 45 mmHg). The patient received an empirical antibiotic treatment with meropenem 1 g q8h, linezolid 600 mg q12h, and caspofungin 50 mg qd, with no substantial benefit. After two weeks, she was still febrile with peaks at 39 °C, and there was an increase in inflammatory markers, with WBC 12,080 cells/mm^3^, C reactive protein (CRP) 12.9 mg/dL, and erythrocyte sedimentation rate (ESR) 78 mm/h. Therefore, the patient was transferred to our National Institute for Infectious Diseases.

Her medical history included hypertension and type 2 diabetes, for which she was taking irbesartan and metformin. She had no other comorbidities. She was a non-smoker and did not drink alcohol. She lived in a rural zone of the Campania region in the south of Italy and occasionally ate homemade, unpasteurized milk products. On admission, she was febrile (37.2 °C), had a respiratory rate of 20 breaths/minute, and had a 93% peripheral oxygen saturation on room air. Arterial pO_2_ was 65 mmHg on room air. The blood pressure was 120/70 mmHg, and the heart rate was 70 beats/minute. Physical examination revealed pale skin and mucosae. Sparse rales were audible in both lungs. No cardiac murmurs were audible. The abdomen was soft, and the liver and spleen were not enlarged. No signs of peripheral embolization, such as petechiae, Roth spots, or Janeway lesions, were present. WBC were 10,710 cells/mm^3^ with 63% neutrophils. Hemoglobin was 9.3 g/dL. Platelets were 521,000/mm^3^. Fibrinogen was 949 mg/dL (normal range up to 450 mg/dL), ESR was 92 mm/h, and CRP was 7.34 mg/dL. Prothrombin time (INR) was 1.23. Lactate dehydrogenase, glucose, urea, creatinine, electrolytes, alanine aminotransferase, and aspartate aminotransferase were normal. Serological tests for *Mycoplasma pneumoniae, Coxiella burnetii,* and *Chlamydophila pneumoniae* were negative. Urinary antigens for *Legionella pneumoniae* and *Streptococcus pneumoniae* were negative. Autoantibodies (antinuclear, anti-mitochondria, anti-smooth muscle, anti-neutrophil cytoplasmic) were negative. Six blood cultures were negative. A direct examination for acid-fast bacteria was negative in the sputum. Culture of sputum yielded the growth of a few colonies of *Stenotrophomonas maltophilia.* A chest CT scan showed multiple infiltrates of both lungs, some with central excavation, no lymphadenopathy, and neither pleural nor pericardial effusion. A CT scan of the abdomen showed no alterations.

On 14 July, the conditions of the patient were slowly deteriorating, with an undulating fever reaching peaks of 39 °C and sweating. A transthoracic echocardiography showed a motile mass of 20 × 17 mm on the atrial side of the tricuspid ring associated with tricuspid insufficiency and pulmonary hypertension. We started an empirical treatment with daptomycin 500 mg/day, piperacillin/tazobactam 4.5 g q6h, oral linezolid 600 mg q12h, and doxycycline 100 mg q12h. We requested serology tests for *Brucella* spp. On 21 July, Wright assay, ELISA IgG, and ELISA IgM were negative. We then requested a Coombs test for *Brucella*. A chest CT scan showed worsening of the radiologic picture with new opacities in both lungs. Anemia worsened with hemoglobin reaching a nadir of 6.9 g/dL, requiring a blood transfusion. 

On 29 July, the *Brucella* Coombs test was positive at a titer of 1:640. A diagnosis of brucellar tricuspid endocarditis was made according to Duke’s criteria [1], and we changed the treatment accordingly. According to the patient’s weight and renal function, a new regimen with intravenous gentamycin 100 mg q12h, trimethoprim-sulphametoxazole 160/800 mg q8h, oral rifampin 600 mg/day, and doxycycline 100 mg q12h was instituted. After 10 days of treatment, the clinical conditions progressively improved, and the fever subsided. A transesophageal echocardiogram evidenced the absence of masses on the tricuspid valve, with the tricuspid leaflets irregularly thickened. A chest CT scan performed on 11 August showed a marked reduction of all infiltrates. Because of nausea, doxycycline was substituted with minocycline 100 mg q12h. On 22 August, the patient was clinically stable, afebrile, and eupnoic. CRP was 0.86 mg/dL, creatinine was 1.2 mg/dL, and WBC were 6730 cells/mm^3^ with a normal differential count. On the same day, the patient was discharged and referred to a local infectious disease specialist for follow-up. She was instructed to avoid the consumption of raw, unpasteurized milk and dairy products. She continued antibiotic treatment for about 9 months with oral rifampin and minocycline plus levofloxacin (750 mg/day). No follow-up serology was made. At the one-year follow-up visit, the patient was in good clinical condition. The blood count and CRP protein were normal. Creatinine and liver function tests were within the normal range. The chest X-ray and transthoracic echocardiogram showed no signs of a recurrence of endocarditis.

## 3. Discussion

Currently, brucellosis is a rare diagnosis in developed countries. However, in the Mediterranean area, this infection is still present in Turkey, Greece, Albania, Spain, Portugal, and Italy [2]. In Italy, at the end of the Second World War, the incidence of human brucellosis was 20 cases/100,000 inhabitants, with the majority of cases registered in the southern regions of Campania, Apulia, Calabria, and Sicily [3]. Since then, however, the incidence of brucellosis has steadily dropped, with an estimated annual incidence rate in the period 2012–2016 ranging between 0.17 and 0.35 cases/100,000 in the whole country. Italy still has a high annual reporting rate [4], and according to an Italian study, the incidence of brucellosis in Italy is largely underestimated [3]. 

Endocarditis is a rare manifestation of brucellosis, representing 0.7–2.3% of all cases [5], and is associated with severe disease and a high mortality rate. In the early antibiotic era and before the introduction of heart surgery for the treatment of endocarditis, mortality from brucellar endocarditis was more than 80% [6]. In a recent series of 308 cases, however, mortality is still high, at 32.7% with medical treatment alone. This figure drops to 6.7% in patients treated with both antibiotics and heart surgery. In this series, the aortic and/or mitral valve was involved in the vast majority (96%) of cases, with large vegetation and perivalvular abscesses frequently reported [7]. On the contrary, tricuspid brucellar endocarditis is rarely reported, so its clinical characteristics are less known. In the medical literature, we were able to retrieve case reports of tricuspid brucellar endocarditis regarding only nine patients [8,9,10,11,12,13,14]. Dourakis et al. [8] presented a case of a 70-year-old man admitted to the hospital with a 2-week history of fever up to 38.5 °C, back pain, and night sweats. He had a pacemaker/defibrillator implanted 30 days before admission. Serological tests for *Brucella* were negative, while blood cultures grew *Brucella melitensis*. Transesophageal echocardiography showed vegetation on the tricuspid valve, which was mildly regurgitant. The patient was treated with doxycycline (200 mg/day), ciprofloxacin (1000 mg/day), and rifampin (900 mg/day) for a scheduled period of 12 months, and the pacemaker/defibrillator was surgically removed. He was completely asymptomatic at the 9-month follow-up. Yazici and colleagues [9] reported the case of a 62-year-old woman admitted to the emergency department complaining of fever, weakness, and swelling of the legs over the last month. Echocardiography revealed enlarged right heart cavities, severe tricuspid regurgitation, and a mass on the tricuspid valve. Blood cultures were negative. The *Brucella* antibody titer was 1:640. The patient was treated with doxycycline 200 mg/day, rifampin 600 mg/day, and ceftriaxone 2 × 2 g IV. After four weeks of treatment, ceftriaxone was discontinued, and the doxycycline plus rifampin treatment was continued for 8 weeks. The patient was asymptomatic at the 6-month follow-up. Rheda et al. [10] reported a case of a 15-year-old boy presenting with fever and dyspnea. A transthoracic echocardiogram showed vegetation of the tricuspid valve and left pulmonary artery with severe tricuspid insufficiency (ejection fraction 46%; PAPs 50 mmHg). Blood cultures and serology were positive for *Brucella melitensis.* The patient was operated on with excision of tricuspid and pulmonary vegetation and tricuspid repair under cardiopulmonary bypass. No details are given on antibiotic treatment. After surgery, the ejection fraction rose to 65%. A favorable medium-term outcome is reported along with medical treatment. Cafardi and colleagues [11] described the case of a 24-year-old woman with a history of injection heroin use, previous tricuspid valve replacement following *S. aureus* endocarditis, and hepatitis C infection, admitted with fever. Multiple blood cultures yielded no growth, and transesophageal echocardiography showed vegetation on the tricuspid prosthesis. Serologic tests for *Brucella* demonstrated a positive IgM, a negative IgG, and a positive serum agglutination test at 1:640. The patient was discharged on oral doxycycline and rifampin as outpatient parenteral therapy with gentamicin was judged to be unacceptably high risk, but after a visit 2 months after discharge, she was lost at follow-up. Jia and colleagues [12] reported 10 cases of *Brucella* endocarditis from China, among which was a fatal case with involvement of the aortic valve, mitral valve, and tricuspid valve. Another case of tricuspid valve brucellar endocarditis was reported by Su et al. [13] from China, but no further information in English was available. Finally, Koruk and colleagues [14] reported 53 patients with *Brucella* endocarditis from Turkey. Among them, three had tricuspid valve endocarditis; all survived, one with medical therapy alone and two with medical and surgical combined therapy. More detailed information about these three cases was not available. The characteristics of the cases described in sufficient detail are summarized in Table 1.

The diagnosis of *Brucella* infection relies on blood cultures and serological tests. As blood cultures are positive only in 40–70% of cases [15], serology is often used for the diagnosis. Several serological tests have been developed, the most commonly used being the classical Wright agglutination test, the Rosa Bengal test, the indirect Coombs test, and the ELISA test. None of these are 100% specific and sensitive, so generally, more than one test is required to establish a diagnosis. In our case, the patient had negative Wright and ELISA testing but a positive Coombs test with a high titer (1:640), making the diagnosis of brucellosis very likely. Moreover, the case fulfilled Duke’s criteria for definite endocarditis [1]. Since the rate of isolation of *Brucella* from blood and other sites varies widely, serological tests are commonly used in brucellosis diagnosis. Many tests are available, each with a different sensibility and specificity, and in cases of suspected brucellosis, more than one test is required for diagnosis. In chronic cases, blocking antibodies of the IgG and IgA classes are present and can block agglutination. In these cases, a Coombs test is used and is frequently positive when the classic agglutination test is negative. Given these facts and the chronic nature of our case, the positivity of the Coombs test as the sole marker of infection was not unexpected [16,17].

Therapeutic schemes for brucellar endocarditis are derived mostly from the experience of single authors and centers in the absence of comparative trials. Treatment is generally based on prolonged courses of combinations of antibiotics active on *Brucella* species and early surgery.

Surgery is usually necessary in left-sided brucellar endocarditis to achieve a cure. Tricuspid endocarditis could be an exception. Among the cases we retrieved from the literature, only one unoperated patient died, but he also had concomitant aortic and mitral endocarditis; four cases recovered with medical therapy only, and three with medical and surgical combined treatment; one patient was lost at follow-up. In our patient, the cardiosurgeon did not give an indication for surgery because of the absence of hemodynamic instability and the very favorable rapid response to antibiotic treatment. 

## 4. Conclusions

Tricuspid brucellar endocarditis is a very uncommon disease, but it should be suspected in a patient living in an endemic area who has a fever and recurrent, unexplained pulmonary embolization. 

Prolonged antibiotic treatment with a combination of drugs active on intracellular microorganisms in the absence of surgical treatment could be effective when the valve is not severely damaged.

## Figures and Tables

**Table 1:** Characteristics and outcome of six cases of brucellar tricuspid endocarditis.

Age	Sex	Comorbidity	Country, Year	Serology	Blood Cultures	Therapy (Months)	Surgery	Outcome	Ref.
70	M	Pacemaker/defibrillator	Greece, 2007	Negative	Positive	Doxi, Rif, Cipro (12)	PMK/DEF removal	Cured	[8]
62	F	NR	Turkey, 2011	1:640	Negative	Doxi, Rif, Cef (3)	No	Cured	[9]
15	M	None	Algeria, 2016	Positive	Positive	NR	Valve repair	Cured	[10]
24	F	IVDU, previous TVR, HCV+	USA, 2019	SAT 1:640	Negative	Doxi, Rif, Lost at follow-up	No	Lost at follow-up	[11]
NR	M	Concomitant aortic and mitral endocarditis	China, 2017	Wright 1:200	Negative	NR	No	Dead	[12]
72	F	AH, diabetes	Italy, 2017	Coombs 1:640	Negative	TMP/SMX, Doxi, Rif, Levo (9)	No	Cured	Our case

AH = arterial hypertension; PMK/DEF = pacemaker/defibrillator; TMP/SMX = trimethoprim/sulphamethoxazole; Doxi = doxycycline; Rif = rifampin; Cef = ceftriaxone; Cipro = ciprofloxacin; Levo = levofloxacin; SAT = serum agglutination test; IVDU =intravenous drug user; TVR = tricuspid valve replacement; NR = not reported.

## Data Availability

Data is unavailable due to privacy restrictions.
